# Subcellular Localization and Cell Cycle-Associated Expression of Human dUTPase Isoforms

**DOI:** 10.3390/biom16101385

**Published:** 2026-09-23

**Authors:** Otília Tóth, Gergely Attila Rácz, Eszter Oláh, Máté Tóth, Edit Szabó, György Várady, Gergely Róna, Beáta G. Vértessy, Nikolett Nagy

**Affiliations:** 1 Department of Applied Biotechnology and Food Sciences, Faculty of Chemical Technology and Biotechnology, BME Budapest University of Technology and Economics, Műegyetem rkp. 3, H-1111 Budapest, Hungary; toth.otilia@ttk.hu (O.T.);; 2 Institute of Molecular Life Sciences, HUN-REN Research Centre for Natural Sciences, Magyar Tudósok Körútja 2, H-1117 Budapest, Hungary; 3 Doctoral School of Biology, Institute of Biology, ELTE Eötvös Loránd University, Pázmány Péter sétány 1/C, H-1117 Budapest, Hungary; 4 Department of Biochemistry and Molecular Pharmacology, NYU Grossman School of Medicine, New York, NY 10016, USA

**Keywords:** cell cycle, subcellular localization, mRNA expression, dUTPase, isoforms

## Abstract

The enzyme dUTPase plays a critical role in maintaining genomic integrity by preventing the misincorporation of uracil into DNA. Beyond the well-described nuclear (DUT-N) and mitochondrial (DUT-M) isoforms, two novel dUTPase isoforms (DUT-3 and DUT-4) have recently been identified in human cells; however, their subcellular localization and cell cycle-associated transcription profiles have not yet been characterized. Here, we demonstrate the localization patterns of all dUTPase isoforms in HCT116 cells, identifying the predominantly cytoplasmic DUT-3 and nuclear DUT-4, as well as the nuclear DUT-N and mitochondrial DUT-M. Moreover, we characterized the endogenous cell cycle-associated isoform-specific expression of dUTPase in human leukemia cell lines U937 and MOLT4. DUT-N exhibits the highest expression and is elevated during the G1/S transition, while DUT-M displays a similar expression pattern at lower levels. The novel DUT-3 isoform shows moderate changes, while DUT-4 shows low constitutive expression during the cell cycle. Our findings support the necessity of dUTPase availability throughout the cell cycle.

## 1. Introduction

Deoxyuridine triphosphate nucleotidohydrolase (dUTPase) catalyzes the hydrolysis of dUTP to dUMP, thereby preventing the misincorporation of uracil into the genome [1]. The resulting dUMP serves as a substrate for the *de novo* thymidylate biosynthesis, thereby maintaining a low intracellular dUTP/dTTP ratio. In the absence of dUTPase activity, dUTP levels increase; however, in the presence of normal DNA repair mechanisms, dUTPase loss does not result in DNA damage or cell death, since misincorporated uracil bases are repaired primarily by the base excision repair (BER) uracil-DNA glycosylases, UNG and SMUG1 [2,3,4]. Human dUTPase is encoded by the *DUT* gene, which gives rise to four distinct isoforms through alternative splicing and alternative promoter usage. Initially, two dUTPase isoforms were described in human cells: a mitochondrial (DUT-M) and a nuclear (DUT-N) isoform [5,6]. Recently, two novel isoforms were identified, presumably a cytoplasmic (DUT-3) and another nuclear (DUT-4) isoform [7].

DUT-M has a mitochondrial target sequence (MTS) on its N-terminal region, which is cleaved upon mitochondrial import [8]. DUT-N predominantly localizes to the nucleus, mediated by an N-terminal nuclear localization signal (NLS) that interacts with importin-α [9], which is also present in DUT-M. Notably, phosphorylation of DUT-N at the Ser11 residue during G2/M phase disrupts this interaction, resulting in the exclusion of DUT-N from the nucleus [9]. Regarding the novel isoforms, DUT-3 does not contain any recognizable subcellular localization signal, while DUT-4 possesses an N-terminal NLS and differs from the nuclear isoform at its N-terminal region. It was previously demonstrated at both the mRNA and protein levels that DUT-N is the predominant isoform with the highest expression, while DUT-M and DUT-3 are expressed at lower, comparable levels [6,7]. For the DUT-4 isoform, only the mRNA expression level was determined, and it was the lowest among all isoforms [7].

It has previously been demonstrated that the promoter regulating DUT-N transcription is highly sensitive to starvation-induced stress, leading to decreased mRNA and protein levels [6,7]. However, the expression of DUT-M and DUT-3 is regulated by a distinct shared promoter; their mRNA levels markedly increased in HeLa, A549, and MDA-MB-231 human cell lines upon serum starvation [7], in contrast to earlier findings in 34Lu human fibroblast cells, where DUT-M expression was reported to be constitutive and independent of the cell cycle [6]. The DUT-4 isoform is driven by an independent promoter and exhibits low constitutive expression upon serum starvation [7]. While mRNA expression of dUTPase isoforms was investigated in detail under serum-starvation conditions, their expression across other cell cycle phases has not yet been characterized. Moreover, the subcellular localization of the novel isoforms was only inferred and has not yet been confirmed.

Therefore, in this study, we investigated the subcellular localization of all dUTPase isoforms by overexpressing them as fusion proteins with a fluorescent eGFP tag, thereby providing insights into their spatial distribution within cells and throughout the different cell cycle phases (G0/G1, S, and G2/M). Furthermore, we analyzed isoform-specific expression of dUTPase in MOLT4 and U937 human leukemia cell lines throughout the cell cycle using a previously described highly robust experimental approach [10]. Investigating the cell cycle is particularly important, as numerous intracellular processes are tightly regulated in a cell cycle-dependent manner [11,12,13,14,15,16]. Moreover, gene and protein expression programs are tightly synchronized with cell cycle progression [17,18,19,20], and perturbation of these regulatory networks can drive malignant transformation and cancer development [21,22,23,24,25]. Notably, dUTPase expression is markedly elevated in certain cancers, especially metastatic colorectal tumors [26,27]. Together, our results contribute to a better understanding of the functional roles of dUTPase isoforms in a cell cycle-dependent context.

## 2. Materials and Methods

### 2.1. Cell Maintenance

MOLT4 is a human adult acute T-lymphoblastic leukemia cell line obtained as part of the NCI-60 human tumor cell line panel from the National Cancer Institute (NCI) Developmental Therapeutics Program (DTP), National Institutes of Health (NIH). U937 is a human adult monocytic lymphoma cell line obtained from ATCC (CRL-1593.2), and HCT116 is a human colorectal cancer cell line obtained from ATCC (CCL-247). MOLT4 and U937 cells were maintained in RPMI-1640 medium with L-glutamine and sodium bicarbonate (Sigma, St. Louis, MO, USA, R8758), while HCT116 cells were maintained in DMEM high glucose with GlutaMAX (Gibco, Waltham, MA, USA, 10564011) supplemented with 1% penicillin-streptomycin (Gibco, Waltham, MA, USA, 15140-122) and 10% fetal bovine serum (Gibco, Waltham, MA, USA, 10500064). All cell lines were maintained in a humidified incubator (Galaxy 170 R, Eppendorf, Hamburg, Germany) at 37 °C with a 5% CO_2_ atmosphere. The absence of mycoplasma infection was verified by genomic DNA isolation and PCR analysis.

### 2.2. Determining the Mitochondrial Co-Localization of DUT-M with Mitotracker

Cells were seeded on ibi-Treat microscopy slides (µ-Slide 8 Well high, 80806, ibidi GmbH, Gräfelfing, Germany) and transfected with 250 ng eGFP-tagged DUT-M plasmid and 1.5 μL XtremeGene HP reagent (Roche, Mannheim, Germany, XTGHP-RO) for 24 h according to the manufacturer’s instructions. Cells were treated with 250 nM Mitotracker (Invitrogen, Eugene, OR, USA, M46752) for 30 min prior to fixation. Cells were washed with PBS and fixed with 4% paraformaldehyde (PFA) in PBS for 15 min at 37 °C, and then, after a 2 × 5 min PBS wash, cells were mounted in Fluoroshield with DAPI (Sigma, St. Louis, MO, USA, F6057). For microscopy analysis, a Leica SP8 confocal microscope (Leica Microsystems GmbH, Wetzlar, Germany) was used. Images from two independent transfections were analyzed using CellProfiler 4.2.8 [28]. Nuclei were segmented from DAPI images (IdentifyPrimaryObjects, Otsu threshold), whole-cell boundaries from transmitted-light images (IdentifySecondaryObjects, propagation), and cytoplasmic regions were obtained by subtracting nuclei from whole-cell objects (IdentifyTertiaryObjects). Manders’ colocalization coefficients (M1 and M2) were calculated per cytoplasmic object using CellProfiler’s MeasureColocalization module. To assess random overlap, one channel was block-scrambled (8 × 8 pixels), and each coefficient was recalculated after 500 independent randomizations, while preserving local pixel structure and intensity statistics. Results supported the conclusion that the observed co-localization reflects spatial association rather than random overlap within the cytoplasm. Analysis was restricted to transfected cells (mean cytoplasmic eGFP intensity > 0.05; *n* = 223 cells).

### 2.3. Generation of Plasmids Encoding eGFP-Tagged dUTPase Isoforms

Plasmid constructs of human DUT-N, DUT-M, and DUT-4 with a C-terminal eGFP tag were purchased from GeneUniversal (Newark, DE, USA) in the eGFP-N1 vector. DUT-3 isoform was generated from the DUT-N plasmid construct using the KLD kit according to the manufacturer’s instructions (New England Biolabs, Ipswich, MA, USA, M0554S) by deleting the differing N-terminal region of DUT-N, thereby generating the protein-coding sequence of DUT-3. To verify the generated DUT-3 plasmid, we sent the purified plasmid for Sanger sequencing and used the verified plasmid for further experiments. Coding sequences of DUT isoforms and primers for the generation of DUT-3 are shown in Supplementary Tables S1 and S2.

### 2.4. Sample Preparation for Image Analysis

Cells were seeded on ibi-Treat microscopy slides (µ-Slide 8 Well high, 80806) at 70% confluency and transfected with 250 ng DNA and 1.5 μL XtremeGene HP reagent (Roche, Mannheim, Germany, XTGHP-RO) for 24 h according to the manufacturer’s instructions. After transfection, cells were treated with 10 µM 5-ethynyl-2′-deoxyuridine (EdU) for 20 min to label cells in S phase. Cells were washed with PBS and fixed with 4% PFA in PBS for 15 min at 37 °C. Cells were blocked and permeabilized with 0.1% Triton-X 100 in 2% BSA PBS for 1 h. After washing cells 2 × 5 min with PBS, the EdU click-it reaction was performed using the Click-iT™ Plus EdU Alexa Fluor™ 647 Flow Cytometry Assay Kit (Invitrogen, Eugene, OR, USA, C10634) according to the manufacturer’s instructions. After 2 × 5 min PBS washes, cells were stained with a cyclin A antibody (Santa Cruz, Dallas, TX, USA, sc-271682) diluted 1:1000 in 2% BSA PBS for 1 h and then washed with PBS for 2 × 5 min. After washing, cells were incubated with Alexa Fluor 546-conjugated anti-mouse secondary antibody (Thermo Fisher Scientific, Waltham, MA, USA, A-11030) for 1 h, then washed with PBS and mounted in Fluoroshield with DAPI (Sigma, St. Louis, MO, USA, F6057). For microscopy analysis, a Leica SP8 confocal microscope was used. Confocal images were acquired using a 63× oil-immersion objective with a pinhole size of 1 Airy unit (AU) for each channel. Fluorescence was detected using photomultiplier tube (PMT) detectors. DAPI and EdU-647 were acquired within the same acquisition sequence but using separate detection windows, while eGFP and Alexa Fluor 546 signals were excited and detected separately to minimize spectral crosstalk. Images were acquired as single optical sections using identical acquisition settings across experimental groups. To determine the subcellular localization of dUTPase isoforms, images from four independent transfections were analyzed. To assess the cell cycle-associated localization of dUTPase isoforms, images from three independent transfections were analyzed.

### 2.5. Image Analysis to Determine the Localization of Human dUTPase Isoforms

Fluorescence confocal microscopy images of eGFP-labeled human dUTPase isoforms and eGFP control cells were analyzed using CellProfiler 4.2.8 [28]. Images were acquired as 8-bit files, and intensities were rescaled to the 0–1 range in CellProfiler. Nuclei, whole cells, and cytoplasm were segmented as separate objects, and mean fluorescence intensities were measured for: mean intensity of eGFP in the nucleus, cytoplasm, and whole cell; EdU mean intensity in the nucleus; and mean intensity of cyclin A in the whole cell. Object-level measurements were exported to CSV files (MyExpt_Nuclei.csv, MyExpt_Cell.csv, MyExpt_Cytoplasm.csv). Per-cell measurements were combined into a single table using a custom Python script (cellprofiler.py). Nuclei, cells, and cytoplasm were linked by parent–child object relationships. For each cell, the following values were used for further analysis: mean and maximal eGFP intensity in nucleus, cytoplasm, and whole cell, nuclear/cytoplasmic eGFP mean intensity ratio, nuclear EdU mean and maximal intensity, and whole-cell cyclin A mean and maximal intensity. The maximum eGFP pixel intensity per cell in the nucleus, cytoplasm, and whole-cell compartments (Intensity_MaxIntensity_GFP) was calculated, and any cell in which at least one compartment reached the maximum intensity defined as ≥0.99 was excluded from further analysis to eliminate cells with saturated pixels. This was applied to eGFP (nucleus, cytoplasm, and whole cell), and to EdU (nucleus) and cyclin A (whole cell) for cell cycle classification. After generating the merged dataset, only eGFP-positive cells were retained for downstream analysis. Cells were filtered if the mean eGFP intensity was >0.05 in at least one of the following compartments: nucleus, cytoplasm, or whole cell. To reduce the inclusion of mitotic cells in the analysis, a nuclear diameter range of 25–60 pixels was applied in CellProfiler; however, this does not completely exclude mitotic cells, which may therefore remain in the designated G0/G1 population. As mitotic cells constitute only a small fraction of the total population, their residual inclusion is expected to have a limited impact on the overall localization analysis. Cell cycle phases were assigned based on nuclear EdU intensity, as a marker of DNA synthesis, and whole-cell cyclin A intensity. EdU-positive cells were classified as S phase, whereas EdU-negative cells were classified as G0/G1 or G2/M based on cyclin A intensity, with a threshold of 0.04 separating the predominant low cyclin A G0/G1 population from high cyclin A G2/M cells. The same threshold with the same mean intensity was applied to EdU intensity to distinguish EdU-negative and EdU-positive cells, based on the bimodal distribution of EdU intensity. Under these criteria, the number of cells filtered in each cell cycle phase is shown on each graph as n. Then, nuclear/cytoplasmic eGFP mean-intensity ratios were plotted by cell cycle phase as boxplots, with data points representing individual cells. A horizontal dashed reference line at a ratio of 1.33 indicated the median nuclear/cytoplasmic eGFP mean intensity ratio of eGFP control cells across all phases, defining a homogeneous distribution baseline for comparison of nuclear or cytoplasmic enrichment.

### 2.6. Cell Synchronization

A total of 2 × 10^7^ cells were seeded into 6-well plates (Corning Inc., Corning, NY, USA Costar) at a density of 2 × 10^6^ cells per well and synchronized with 10 μM RO-3306 (Sigma, St. Louis, MO, USA, SML0569) reagent for 20 h. Then, the cells were centrifuged at 150× *g* for 5 min and washed with PBS (Sigma, St. Louis, MO, USA, P3813). Cells were resuspended in fresh medium, divided into 2 million cells, and transferred to T25 flasks. Samples were collected every 2 h (U937) and every 4 h (MOLT4) for 16 and 32 h, respectively. Before every sample collection, cells were treated with 10 μM EdU for 20 min. Cells were centrifuged at 200× *g* for 5 min and washed with PBS. Cells were divided into two groups for qPCR analysis and to determine the cell cycle phase distribution.

### 2.7. Determination of Cell Cycle Phase Distribution

To analyze the distribution of cells across cell cycle phases, we used the Click-iT™ Plus EdU Alexa Fluor™ 488 Flow Cytometry Assay Kit (Invitrogen, Eugene, OR, USA, C10632). Cells were treated with 10 μM EdU for 20 min, then collected and washed with 1% BSA diluted in PBS. Cells were centrifuged at 200× *g* for 5 min, then fixed using 4% PFA diluted in PBS for 15 min. Cells were centrifuged at 200× *g* for 5 min, resuspended in 1 mL of 1% BSA in PBS, and stored at 4 °C until further use. Permeabilization and the click-it reaction were performed according to the manufacturer’s recommendation. After the reaction, cells were incubated with 10 μg/mL propidium iodide and 20 μg/mL RNase A diluted in PBS for 30 min at room temperature, protected from light. An Attune NxT flow cytometer (Thermo Fisher Scientific, Waltham, MA, USA) was used to analyze the cell cycle. The Click-iT™ Plus EdU Alexa Fluor™ 488 signal was detected with 488 nm excitation and 530/30 nm emission in the BL1 channel. Propidium iodide signal was detected with 488 nm excitation and 695/40 nm emission in the BL3 channel. Data analysis was conducted using Attune NxT software version 3.2.1. Data on cell ratios in each phase and at each time point are available in Supplementary Figure S1 of our previous work [10].

### 2.8. RNA Isolation and RT-qPCR

Cells were collected, centrifuged at 200× *g* for 4 min, and washed with PBS. Cell pellets were extracted by vortexing the cells with 600 μL RLT buffer (Qiagen, Hilden, Germany, RNeasy Mini Kit, 74104) supplemented with β-mercaptoethanol and glass beads for 1 min. Samples were stored at −20 °C until further use. For RNA isolation, we used the Qiagen RNeasy Mini Kit (Cat: 74104) according to the manufacturer’s recommendations. DNase treatment was also performed according to the manufacturer’s recommendations (Qiagen, Hilden, Germany, RNase-Free DNase Set, 79254). We verified the purity of the isolated RNA using Nanodrop 2000 (Thermo Fisher Scientific, Waltham, MA, USA), while the integrity of the samples was tested using 1% TBE agarose gel electrophoresis. The concentrations, purities, and agarose gels of isolated RNA samples are shown in Supplementary Figure S2 and Table S3 of our previous work [10]. 200 ng RNA was transcribed into cDNA using the Maxima First Strand cDNA Synthesis Kit for RT-qPCR (Thermo Scientific, Waltham, MA, USA, K1641) at 65 °C for 30 min. The qPCR measurements were performed using the previously described optimized RT-qPCR method [7,10]. The sequences of the selected primer pairs are available in the Supplementary Table S3. For each target, three biological replicates and three technical replicates were measured in every sample collected at each time point. To ensure reagent purity, three replicates of no template controls (NTC) were used for each measurement and target. To verify the absence of genomic DNA contamination, no reverse transcriptase (NRT) controls were prepared, and random measurements were performed on 35% of the samples. The NRT samples were generated for each RNA sample in the absence of the RT enzyme. The differences between Cq values of NRT/NTC controls and the samples were greater than 10 in most cases and greater than 5 in all cases. For RT-qPCR analysis, Clear Hard-Shell 96-Well PCR Plates (Bio-Rad, Hercules, CA, USA) and Microseal ‘B’ PCR Plate Sealing Film (Bio-Rad) were used. Thermal cycling and detection were performed in the CFX96 real-time PCR detection system (Bio-Rad). The thermal cycling protocol included initial denaturation and hot-start polymerase activation using MyTaq HS mix (Bioline GmbH, Luckenwalde, Germany) at 95 °C for 5 min, followed by 50 cycles of denaturation at 95 °C for 30 s and annealing/extension at 63 °C for 30 s. After each measurement, melting curve analysis was routinely performed from 60 °C to 95 °C with a 0.5 °C increment every 5 s to assess PCR product specificity. If the melting curves showed a specific product formation, the wells were excluded from the analysis. Relative expression data were calculated using CFX Maestro 2.0 software (Bio-Rad, Hercules, CA, USA), accounting for PCR efficiency for each target.

### 2.9. Data Analysis of qPCR

Measured Cq values for all DUT targets were corrected based on a separate qPCR assay performed using PCR products of DUT-M, DUT-N, DUT-3, and DUT-4 targets at a final concentration of 0.01 and 10 fg/μL. Based on the differences between the Cq values measured for all *DUT* targets at the same concentration, correction factors were determined for each target (Supplementary Table S4). Measured Cq values of technical and biological replicates of *DUT* targets corresponding to each time point were adjusted using these correction factors (Supplementary Tables S5 and S6).

Relative normalized expression values were calculated using CFX Maestro 2.0 software (Bio-Rad, Hercules, CA, USA) for each target and time point. For normalization, the reference genes *CNOT4*, *TBP*, and *PUM1* were used for MOLT4, whereas *SNW1*, *TBP*, and *UBC* were applied for the U937 cell line. To correct relative normalized expression values, differences in corrected Cq values were calculated, and (1 + E)^ΔCq^ values were determined considering PCR efficiencies for each target. Using these values, the relative, normalized, corrected expression of dUTPase targets was determined to compare isoform expression within the same cell line (Supplementary Table S7). Upper and lower standard error values were calculated from biological replicates using the same approach (Supplementary Table S7). Finally, the relative, normalized, corrected expression (RNE_corrected_) data were visualized using Origin 2018 (OriginLab Corporation, Northampton, MA, USA). Non-corrected, relative normalized mRNA expression data (RNE) of dUTPase isoforms are shown in Supplementary Figure S1. Graphs display RNE relative to zero for each target.

### 2.10. Western Blot Analysis

Cells were lysed by vortexing for 1 min in RIPA buffer (150 mM NaCl, 1% Nonidet P-40 Substitute, 0.5% sodium deoxycholate, 0.1% SDS, 50 mM Tris-HCl, 2 mM DTT, 1 mM PMSF, 5 mM benzamidine, 2 mM EDTA) supplemented with cOmplete ULTRA Tablets (Roche). Samples were sonicated twice for 3 min with a degas function at 4 °C (Elma S 30 H Elmasonic, Elma Schmidbauer GmbH, Singen, Germany) and centrifuged for 10 min at 4 °C at 10,000× *g* (Eppendorf Centrifuge 5804 R, Eppendorf, Hamburg, Germany). Supernatant was collected and stored at −20 °C for further use. For Western blot analysis, lysates were incubated with loading buffer 5× (250 mM Tris-HCl, 50% glycerol, 10% DTT, 10% SDS, 0.05% bromophenol blue, pH = 6.8) at 95 °C for 5 min and loaded onto a 16% polyacrylamide gel. GRS Protein Marker Multicolor (GRiSP, Porto, Portugal, GLP01.0500) was used as a protein ladder. Electrophoresis was performed in the Mini-PROTEAN Electrophoresis System (Bio-Rad) in SDS Running Buffer (25 mM Tris-HCl, 20 mM glycine, 0.1% SDS) at 200 V for 1 h. For transfer, a PVDF transfer membrane with 0.45 µm pore size (Immobilon-P, Merck, Carrigtwohill, Ireland, IPVH09120) was used in transfer buffer (10 mM CAPS, 15% methanol, pH = 11) at 250 mA for 3 h. Membranes were cut after transfer and placed in TBS-T (25 mM Tris-HCl, 140 mM NaCl, 3 mM KCl, 0.05% Tween-20, pH = 7.4). Blocking and immunostaining were performed in 5% skimmed milk powder in TBS-T for 1 h. After blocking, membranes were incubated with primary antibodies overnight: anti-dUTPase antibody produced in rabbit (Huabio, Hangzhou, China, HA721389), anti-cyclin E antibody produced in mouse (Santa Cruz, Dallas, TX, USA, sc-247), anti-actin antibody produced in rabbit (Sigma, St. Louis, MO, USA, A5060), anti-eGFP antibody produced in rabbit (Invitrogen, Eugene, OR, USA, A11122), and anti-vinculin antibody produced in rabbit (Bethyl, Montgomery, TX, USA, A302-534A). After washing membranes with TBS-T for 3 × 10 min, incubation with HRP-conjugated secondary antibodies was performed for 2 h at RT, protected from light (anti-rabbit IgG (GE Healthcare, Munich, Germany, Na934vs) for dUTPase, actin, and eGFP, and anti-mouse IgG (Sigma, St. Louis, MO, USA, A9044) for cyclin E). After washing membranes with TBS-T for 3 × 10 min, membranes were placed in TBS buffer until imaging. Bands were visualized using Immobilon Western Chemiluminescent HRP Substrate (Merck, Carrigtwohill, Ireland, WBKLS0100). Imaging was performed with the ChemiDoc MP Imaging System (Bio-Rad, Hercules, CA, USA). Uncropped Western blot images are shown in Supplementary Figures S2 and S3.

### 2.11. Software Used in This Study

Microscopic images were acquired using Leica LAS X software (Leica Microsystems GmbH, Wetzlar, Germany, v3.10.0). Post-processing and image scaling were performed using Fiji (https://fiji.sc/) (accessed on 27 July 2026). For image analysis, CellProfiler was used [28]. Image analysis, CSV sheet handling, editing, and the generation of Python scripts, including those used to generate boxplot visualizations, were performed using Cursor with assistance from generative artificial intelligence. Relative expression data were calculated using CFX Maestro 2.0 software (Bio-Rad, Hercules, CA, USA). Graphs of mRNA expression were generated using Origin 2018 (OriginLab Corporation, Northampton, MA, USA, v b9.5.1.195). Western blot images were created with Image Lab 4.1 software (Bio-Rad, Hercules, CA, USA). Schematic figures were created in BioRender: https://BioRender.com. Final figures were assembled using Adobe Illustrator CC 2020 (Adobe Inc., San Jose, CA, USA, v25.3.1).

## 3. Results and Discussion

### 3.1. Alternative Splicing of Human dUTPase Isoforms

The enzyme dUTPase is encoded by a single gene, which gives rise to four different isoforms through alternative splicing (Figure 1). These include the previously characterized nuclear, DUT-N, and mitochondrial, DUT-M, as well as the recently identified DUT-3 and DUT-4 isoforms. All isoforms differ in their N-terminal regions, encoded by exons 1–3, whereas their C-terminal regions are identical and encoded by exons 4–8 (Figure 1A,B). As a result of alternative splicing and differences in the 5′ ends of dUTPase transcripts, isoform-specific mRNA expression can be selectively determined by RT-qPCR analysis using an appropriately designed method (Figure 1A,B) [7]. The longest isoform, DUT-M, contains an N-terminal MTS encoded by exon 2, and during alternative splicing, the 5′ region of exon 3 is skipped. The shortest isoform, DUT-3, is transcribed from the same promoter as DUT-M, contains the 5′ region of exon 2, skipping the MTS-encoding region, and, like DUT-M, skips the 5′ region of exon 3. The precursor form of DUT-M containing the MTS is proteolytically cleaved into a lower-molecular-weight mature form upon import into the mitochondria, as demonstrated previously [6,8] (Figure 1C,D). DUT-3 is translated from a downstream initiation site located within exon 3, resulting in the absence of any known subcellular localization signals (Figure 1C,D). Transcription of the DUT-N isoform starts with exon 3, exclusively including the 5′ part of exon 3 containing its specific translation initiation site (MPCSE) (Figure 1D). DUT-4 is the only isoform containing exon 1, thereby resulting in eight amino acids difference (MAAGGAAP) from DUT-N (Figure 1D). All isoforms except DUT-3 contain an NLS (KRAR) [8] and potential phosphorylation sites [9] (Figure 1C,D).

### 3.2. Subcellular Localization of Human dUTPase Isoforms in HCT116 Cells

Previously, the nuclear localization of DUT-N and the mitochondrial localization of DUT-M were identified using fluorescence microscopy and immunohistochemistry [8]. In this study, we aimed to determine the subcellular localization of the recently identified isoforms, DUT-3 and DUT-4, as well as DUT-N and DUT-M. Since dUTPase isoforms differ only in their N-terminal regions, and the sequence of DUT-3 is otherwise identical to that of the other isoforms, commercially available antibodies, which are generally directed against the regions common to all isoforms, cannot discriminate between individual dUTPase isoforms. However, isoform-specific custom antibodies could be generated against the unique MTS “ASTVGAAGWKGELPKAGGSPAPGP” of the mature form of DUT-M [6,8], or the unique “MPCSE” or “MAAGGAAP” sequences in DUT-N and DUT-4, respectively. Even with such antibodies, isoform-specific immunostaining of dUTPase could not be achieved, as the DUT-3 isoform lacks any distinctive sequence. Therefore, isoform-specific subcellular localization of dUTPase can only be determined using fluorescence-labeled protein constructs. Accordingly, in our study, dUTPase isoforms were expressed in human colorectal HCT116 cells as eGFP fusion proteins in the eGFP-N1 vector with a C-terminal eGFP tag for each dUTPase isoform to enable fluorescence-based detection. Following transient transfection of HCT116 cells, the subcellular localization of the eGFP-tagged dUTPase isoforms was analyzed by confocal microscopy. As a control, an unfused eGFP protein in the eGFP-N1 vector was used. Figure 2A shows representative images of the subcellular localization of the eGFP control and dUTPase isoforms. In agreement with previous observations, DUT-N shows predominantly nuclear localization consistent with the presence of an N-terminal NLS, while DUT-M shows a punctate pattern in the cytoplasm, suggesting mitochondrial localization [8]. DUT-3 was observed to reside primarily in the cytoplasm, as expected given the absence of any known subcellular localization signals. DUT-4 localization was detected mainly in the nucleus, in agreement with the presence of the N-terminal NLS sequence. However, overexpression of eGFP-tagged proteins may affect the localization patterns of the investigated proteins. Although the localization of eGFP-labeled DUT-M has been described previously, its pattern was not validated by co-localization with a mitochondrial marker [8]. Therefore, we applied Mitotracker to visualize mitochondria after transfection with eGFP-tagged DUT-M to validate its mitochondrial localization (Figure 2B). To quantitatively evaluate this co-localization, image analysis was performed on 223 transfected cells using CellProfiler [28] (Figure 2C). Manders’ co-localization coefficients were calculated for each cell, yielding high M1 and M2 values (mean M1 = 0.783 and M2 = 0.527), indicating strong overlap between the fluorescent signals of DUT-M eGFP and Mitotracker, providing quantitative support for the mitochondrial localization of DUT-M.

To quantitatively compare the subcellular distribution of eGFP-labeled dUTPase isoforms, the mean intensity of the eGFP fluorescent signal was determined separately in the nucleus and the cytoplasm for each transfected cell using CellProfiler [28]. The nuclear/cytoplasmic eGFP mean intensity ratio was calculated for each eGFP-positive cell and used as a quantitative measure of subcellular localization. To establish a reference for homogeneous distribution, the nuclear/cytoplasmic eGFP mean intensity ratio was empirically determined in eGFP control cells under our experimental conditions. Although free eGFP is sufficiently small to passively diffuse through the nuclear pore complex, its measured nuclear/cytoplasmic ratio in our assay was 1.33 rather than the theoretical value of 1.0. We therefore used the experimentally determined value of 1.33 as the baseline for assessing the subcellular localization of the eGFP-tagged dUTPase isoforms. Cells with higher nuclear/cytoplasmic eGFP mean intensity ratios suggest nuclear localization, while lower values suggest cytoplasmic localization. In the case of DUT-N and DUT-4, cells exhibit higher ratios than the eGFP control, indicating their nuclear localization (Figure 2D). On the other hand, cells expressing eGFP-labeled DUT-M and DUT-3 show lower ratios compared to the eGFP control, suggesting their cytoplasmic localization. Taken together, these findings indicate that the four dUTPase isoforms exhibit distinct subcellular localization patterns. Specifically, DUT-N and DUT-4 show a predominantly nuclear localization pattern, while DUT-M exhibits mainly mitochondrial localization, and DUT-3 displays primarily cytoplasmic localization.

### 3.3. Cell Cycle-Associated Localization of Human dUTPase Isoforms in HCT116 Cells

To investigate the cell cycle-associated localization of eGFP-labeled human dUTPase isoforms, cells were transfected with each eGFP-tagged dUTPase construct and an empty eGFP control. Cells were treated with the nucleotide analog EdU, to visualize cells in S phase actively replicating DNA, followed by click-it detection and immunostaining for cyclin A, highly expressed from late S to G2/M phase. Cell cycle phases were distinguished based on EdU and cyclin A signal patterns. Cells exhibiting a positive EdU signal were classified as being in S phase, whereas cells positive for cyclin A but negative for EdU were assigned to the G2/M phase. Cells lacking both cyclin A and EdU signals were classified as being in the G0/G1 phase (Figure 3A). Since cyclin A is degraded during prometaphase, late mitotic cells may also be classified as G0/G1 cyclin A-negative cells according to our assessment. To minimize their inclusion, we restricted the nuclear diameter range during image analysis; however, a small fraction of mitotic cells may still remain in the G0/G1 population. Image analysis was performed using CellProfiler to investigate the cell cycle-associated subcellular localization of eGFP-tagged dUTPase isoforms and eGFP control (Figure 3B). Cells transfected with eGFP control show a nuclear/cytoplasmic mean intensity ratio of 1.33 and therefore are used as a baseline to determine the subcellular localization of dUTPase isoforms. Higher nuclear/cytoplasmic eGFP mean intensity ratio values suggest nuclear localization, while lower ratio values suggest cytoplasmic localization. According to our analysis, most cells expressing DUT-N eGFP exhibit higher ratio values than the eGFP control across all investigated cell cycle phases, suggesting nuclear localization.

DUT-M exhibited lower nuclear/cytoplasmic ratio values than the eGFP control across all cell cycle phases, consistent with predominantly cytoplasmic localization. Given the co-localization of DUT-M with Mitotracker (Figure 2B,C), this cytoplasmic signal is largely attributable to its mitochondrial localization. The recently identified isoform DUT-3 shows lower ratios than the eGFP control, indicating its predominantly cytoplasmic localization across all cell cycle phases investigated. The recently identified nuclear isoform, DUT-4, shows a similar localization pattern and ratio values to DUT-N across all cell cycle phases, suggesting its nuclear presence. Although DUT-4 also contains the potential serine phosphorylation site identified in DUT-N and shown to be phosphorylated during mitosis [9], whether DUT-4 undergoes similar mitosis-dependent phosphorylation remains to be elucidated.

To assess the integrity of eGFP-tagged dUTPase constructs, we performed Western blot analysis of HCT116 cells transfected with the C-terminal eGFP-tagged dUTPase isoforms (DUT-N, -M, -3, and -4), eGFP alone, or no construct, using an anti-eGFP antibody (Supplementary Figure S2). In addition to the predominant fusion-protein bands at the expected molecular weights, a small amount of free eGFP was detected in all transfected samples, whereas no eGFP signal was observed in non-transfected cells. For DUT-M eGFP, two weaker bands were detected, consistent with the full-length fusion protein containing the MTS region and the processed form following its cleavage after mitochondrial import. These results confirm the presence of a small amount of free eGFP in the transfected cells, which may contribute to the determined nuclear/cytoplasmic mean intensity ratio values and may introduce a systematic upward shift.

The distinct localization and expression patterns of human dUTPase isoforms may reflect a compartment-specific organization of dUTP sanitization that supports genome integrity in both the nucleus and mitochondria. Similar to other nucleotide-sanitizing enzymes, including DCTPP1, NUDT1, and ITPase, which are predominantly cytoplasmic [29,30,31], the cytoplasmic localization of DUT-3 suggests that control of the cytosolic dUTP pool may be an important component of nucleotide homeostasis. This is particularly relevant since cytoplasmic dNTPs contribute to both nuclear and mitochondrial DNA synthesis, while mitochondrial dNTP pools are additionally sustained by a local salvage pathway [32,33]. In contrast, the mitochondrial localization of DUT-M is consistent with a more direct role in protecting mitochondrial DNA from nucleotide pool imbalances, analogous to other enzymes involved in base excision repair and nucleotide sanitization [32,33,34]. The differential abundance of the isoforms further suggests that their relative contribution may depend on cellular proliferative state. In proliferating cells, DUT-N is the predominant isoform, consistent with a major requirement for dUTP control during active nuclear DNA replication, whereas DUT-M, DUT-3, and DUT-4 are expressed at comparatively low levels. Upon serum starvation, however, DUT-N levels decrease, while DUT-3 and DUT-M increase, with DUT-4 remaining unchanged in human cell lines [7]. This coordinated shift may indicate a redistribution of nucleotide sanitization capacity as cells transition from proliferation to quiescence, when *de novo* dNTP synthesis is reduced, and mitochondrial DNA maintenance relies more heavily on the nucleotide salvage pathway [32,33,34]. Thus, rather than representing redundant isoforms, the distinct dUTPase isoforms may provide complementary layers of dUTP control across cellular compartments and metabolic states, thereby contributing to the maintenance of the integrity of both nuclear and mitochondrial genomes.

### 3.4. Cell Cycle-Associated Expression of Endogenous dUTPase Isoforms in MOLT4 and U937 Human Leukemia Cell Lines

Another aim of this study was to investigate endogenous mRNA expression of each dUTPase isoform throughout the cell cycle, as this has not been systematically addressed in the literature. Isoform-specific dUTPase mRNA expression profiles were determined previously in 7 normal and 13 cancer cell lines [7], among which two suspension leukemia cell lines, MOLT4 and U937, exhibited the highest dUTPase expression levels; therefore, we selected them for analyzing cell cycle-specific mRNA expression. Cells were synchronized using RO-3306 reagent, a cyclin-dependent kinase (CDK1) inhibitor that reversibly arrests cells at the G2/M phase boundary [35]. After releasing the cells from the RO-3306 block, they were collected at specified time points to collect cells enriched in G0/G1, S, and G2/M cell cycle phases.

The distribution of cells across the cell cycle phases, G0/G1, S, and G2/M, was determined by flow cytometry, as described previously [10]. The ratio of cells in each phase was visualized by 100% stacked area diagrams (Figure 4A). Since suspension cell lines were used, complete synchronization in G2/M phase using RO-3306, followed by mitotic shake-off, could not be achieved. However, 4–8 h after release, the majority of cells progressed into G1 phase, and subsequently, both investigated cell lines exhibited robust entry into S phase between 16 and 24 h, indicating successful synchronization. After release, the ratio of G2/M phase cells rapidly decreased, while the population of cells in the G0/G1 phase continuously increased in both cell lines, reaching a maximum at 12 h in MOLT4 and 8 h in U937 cell lines. Subsequently, the G0/G1 population decreased, and the proportion of S-phase cells increased, peaking at 24 h in MOLT4 and 16 h in U937 cells. Although a heterogeneous cell population was analyzed, samples collected at specific time points were enriched for each cell cycle phase, enabling investigation of isoform-specific dUTPase expression throughout the cell cycle in both cell lines.

We have previously demonstrated that the expression of reference genes used for RT-qPCR normalization is strongly influenced not only by experimental conditions and cell line-specific differences [36], but also by the cell cycle [10]. Accordingly, *CNOT4*, *TBP*, and *PUM1* were identified as the most stable reference genes in MOLT4, while *SNW1*, *TBP*, and *UBC* were identified as the most suitable in U937 cells [10]; therefore, these genes were used to normalize cell cycle-specific mRNA expression data. In addition, data analysis was performed to allow direct comparison of the relative normalized expression levels of the different dUTPase isoforms within the same cell line.

Among all isoforms, the nuclear DUT-N exhibited the highest expression levels at all time points investigated, showing 5-fold and 1.9-fold oscillations throughout the cell cycle in MOLT4 and U937 cell lines, respectively (Figure 4B). Moreover, DUT-N showed an approximately 400-fold higher relative normalized corrected expression than the isoform exhibiting the lowest expression, i.e., DUT-4. We observed 2.7-fold oscillations in DUT-3 and DUT-4 in both cell lines throughout the cell cycle. Regarding DUT-M, it exhibits slightly higher expression than DUT-3 and DUT-4 and displays a 3-fold oscillation throughout the cell cycle in both cell lines (Supplementary Table S7). Despite DUT-M and DUT-3 sharing the same promoter, DUT-M shows higher expression and differences during the cell cycle, suggesting an alternate regulatory mechanism.

To determine the cell cycle-associated expression of dUTPase isoforms, cell cycle phase distribution was compared with mRNA expression patterns. In MOLT4 cells, DUT-N expression increased during the G1/S transition from 8 h, reaching its maximum at 20 h (Figure 4A,B). Notably, this peak occurred before the maximal enrichment of S-phase cells, suggesting that DUT-N mRNA expression is upregulated precisely prior to S phase to ensure sufficient protein levels and, therefore, an appropriate dUTP/dTTP ratio necessary for DNA synthesis. In U937 cells, a distinct pattern was observed, reflecting differences in synchronization efficiency. In this cell line, the highest DUT-N expression was detected at 0 h, when both G2/M-phase and S-phase cells accounted for nearly 50% of the population. Furthermore, elevated DUT-N expression was observed at 10–16 h, during the G1/S transition with a continuous increase in the number of S-phase cells, further supporting the S-phase-associated regulation of DUT-N. Notably, more pronounced differences in the expression levels were observed in the MOLT4 cell line than in U937 cells, which may correlate with the twofold longer generation time of MOLT4 cells. The prolonged cell cycle duration provides a broader temporal window over which changes in transcript levels may occur and become apparent following release from synchronization. Consequently, the observed differences in expression patterns between the two cell lines may reflect differences in generation time, cell cycle progression, and temporal regulation of transcript abundance.

Expression of DUT-M was markedly lower in both cell lines compared to DUT-N, with a maximal expression detected at 16–20 h in MOLT4 and at 16 h in U937 cells, corresponding to the G0/G1 phase, and was analogous to the time points at which DUT-N expression also peaked. Although differences across samples were smaller, this temporal overlap suggests that DUT-M expression is also associated with the G1/S transition. This observation is consistent with the literature, as mitochondrial DNA replication occurs primarily during S phase rather than G1 phase [37].

Isoforms DUT-3 and DUT-4 exhibited the lowest expression levels in both cell lines investigated and showed relatively stable expression patterns throughout cell cycle progression, with DUT-3 displaying relatively minor variation across the analyzed time points, suggesting a limited dependence on cell cycle progression. Since it is well established that a substantial dNTP pool is present in the cytoplasm, dUTPase—similarly to other sanitizing enzymes—is localized to the cytoplasm as well, with consistent expression throughout the entire cell cycle. Regarding DUT-4, it exhibits more constitutive expression throughout the cell cycle, consistent with previous findings demonstrating that its expression remains unchanged even under serum starvation conditions [7].

Our results demonstrate that, in both cell lines, DUT-N exhibits an S phase-associated mRNA expression pattern. Moreover, it was demonstrated that DUT-N is an E2F1 target [38], which is known to regulate genes with G1/S-peaking expression patterns [39]. According to our analysis, the maximal mRNA expression of DUT-N was observed during the G1/S phase transition in U937 and MOLT4 human leukemia cell lines.

However, DUT-M expression was described to be constitutive in 34Lu human lung cancer cells [6]; here, we demonstrate that it increases expression during G1/S transition. Moreover, DUT-M was also shown to change upon serum starvation in HeLa, MDA-MB-231, and A549 cells [7], underlining that DUT-M shows cell cycle dependency. Although DUT-3 and DUT-M are driven by the same promoter, DUT-3 shows minor changes and lower expression, suggesting an alternative regulatory mechanism. DUT-4 is expressed at a substantially lower level than the other isoforms, and it is unaffected by cell cycle progression, supporting the nuclear presence of dUTPase independently of the cell cycle. Notably, cell synchronization using RO-3306 increases the proportion of cells in the G2/M phase but does not fully enrich the entire cell population in this phase, particularly in suspension cell lines. Consequently, the release of synchronized cells results in a heterogeneous population containing cells in all cell cycle phases at varying proportions. This heterogeneity may influence the measured mRNA expression levels. However, the primary observations reliably represent the enriched cell cycle phases, and the overall conclusions can be inferred.

To further investigate the protein expression of dUTPase isoforms during the cell cycle, we performed Western blot analyses of U937 and MOLT4 cells (Figure 4C and Figure S3). The cell cycle phase distribution of the collected samples was verified by flow cytometry analysis. The predicted molecular weight of DUT-N is 17.75 kDa, whereas that of DUT-3 is 15.41 kDa. Consistent with these predicted sizes, a distinct band at lower molecular weight was detected in both MOLT4 and U937 cell lines, supporting the presence of DUT-3 at the protein level across the analyzed time points. The predominant band detected at approximately 18 kDa is also consistent with the predicted molecular weight of DUT-N. DUT-4 differs from DUT-N by only eight amino acids and is expressed at a substantially low mRNA level. Given their highly similar molecular weights, DUT-4 cannot be distinguished from DUT-N by conventional Western blotting. For DUT-M, the precursor form retaining the MTS with a predicted molecular weight of 19.35 kDa was detected as a band above the predominant DUT-N band. Following mitochondrial import, the MTS is cleaved, resulting in a mature form with an expected molecular weight overlapping that of DUT-N. Moreover, the MTS-containing precursor is expected to be relatively transient due to cleavage following mitochondrial import. Consequently, the mature DUT-M isoform cannot be distinguished from DUT-N by Western blotting.

To assess cell cycle-associated changes in protein levels, cyclin E was used as an S phase marker. In both cell lines, cyclin E levels increased after release, reaching the highest level at 32 h in MOLT4 and at 16 h in the U937 cell line, consistent with an enrichment of cells in S phase. Notably, the intensity of the 18 kDa band, which is largely attributed to the most abundant isoform, DUT-N, also increased in these samples, suggesting that DUT-N protein levels may be associated with S phase enrichment, consistent with previous studies [6,38]. In contrast, the lower amount of DUT-3 and DUT-M at the protein level limits the sensitivity of Western blot analysis and consequently makes cell cycle-associated changes in these isoforms more difficult to detect. Therefore, while the protein level of the predominant DUT-N isoform supports an S phase-associated pattern, the low expression levels of DUT-3 and DUT-M preclude reliable assessment of their cell cycle-associated regulation by Western blot.

The protein level validation of DUT-4 poses an additional challenge due to its high similarity to DUT-N. The DUT-4-specific N-terminal sequence “MAAGGAAP” is the only difference between the two isoforms, and their similar molecular weights preclude reliable separation by Western blot. Detection of a specific peptide by mass spectrometry would provide a more direct approach for validating DUT-4 protein expression. Importantly, previously published proteomic data included in the ProteomicsDB database provided evidence for the presence of DUT-4 in HCT116 cells by identifying peptides “AAGGAAPETPAISPSKR” and “AAGGAAPETPAISPSK” [40]. Both peptides contain the “AAGGAAP” sequence, which is unique to DUT-4, followed by the common dUTPase sequence. Their identification, therefore, provides proteomic evidence supporting the presence of the DUT-4 isoform in HCT116 cells. Nevertheless, additional targeted LC-MS/MS analyses in independent cellular models will be required to directly validate DUT-4 protein expression and to determine its abundance and cellular distribution.

## 4. Conclusions

In the present study, we characterized the cell cycle-associated subcellular localization and endogenous mRNA expression of human dUTPase isoforms by combining fluorescence-based localization with isoform-specific RT-qPCR analysis across distinct cell cycle phases in different human cell lines. In this study, for localization and microscopy analyses, adherent HCT116 cells were used to identify the subcellular localization of eGFP-tagged dUTPase isoforms. To determine cell cycle-associated endogenous mRNA expression, MOLT4 and U937 suspension cell lines were used because they express high levels of dUTPase and can be readily collected for this assay after synchronization.

To investigate the cell cycle-associated subcellular localization of human dUTPase isoforms, cells were transfected with eGFP-labeled DUT-N, DUT-M, DUT-3, DUT-4, or eGFP control. Cell cycle phases were identified using EdU incorporation to mark S-phase cells and cyclin A immunostaining to distinguish G0/G1 from G2/M phase. Subcellular localization was subsequently assessed by image analysis. Our results confirmed that DUT-N and DUT-4 are predominantly localized in the nucleus, whereas DUT-3 is mainly cytoplasmic. For DUT-M, co-localization with Mitotracker confirmed its previously suggested mitochondrial localization. The similar nuclear localization patterns of DUT-N and DUT-4, together with the presence of the Ser11 residue in DUT-4, raise the possibility of a similar phosphorylation-dependent localization mechanism, although this remains to be elucidated for DUT-4. Our results extend the current understanding of dUTPase regulation and compartmentalization in human cells. These findings indicate that dUTPase isoforms are localized to the nucleus, cytoplasm, and mitochondria. This spatial distribution suggests a compartment-specific organization of dUTP hydrolysis, consistent with the need to maintain genomic integrity in both nuclear and mitochondrial DNA, as well as to sanitize the cytoplasmic dNTP pool.

Cell cycle-associated expression analysis revealed a distinct regulatory pattern among the isoforms. Nuclear DUT-N exhibited the highest expression levels throughout the entire cell cycle and showed higher mRNA expression levels during the G1/S transition in both leukemia cell lines examined, i.e., MOLT4 and U937, supporting its well-established role in maintaining a low dUTP/dTTP ratio. Notably, DUT-M also displayed higher mRNA expression levels during G1/S transition, suggesting coordinated regulation of nuclear and mitochondrial dUTPase activity during DNA synthesis. Importantly, the maximal expression of both DUT-N and DUT-M preceded the highest ratio of S-phase cells, indicating that transcriptional upregulation occurs in advance of S-phase entry during G1/S transition. In contrast, DUT-3 displays minor mRNA expression changes during the cell cycle, while DUT-4 was expressed at substantially lower levels without characteristic cell cycle-dependency. Western blot analysis revealed that the predominant DUT-N isoform exhibits an expression pattern consistent with S-phase association, while DUT-M and DUT-3 isoforms are detectable with lower levels throughout the cell cycle. The minimal molecular weight difference between DUT-4 and DUT-N does not allow us to reliably resolve DUT-4 with this technique; however, the unique peptides of DUT-4 were previously detected using MS/MS analysis supporting its existence at the protein level.

In summary, our findings suggest that dUTPase isoforms are differentially regulated, consistent with their distinct subcellular localizations. The presence of four distinct human dUTPase isoforms may provide functional flexibility by enabling differential regulation of dUTPase expression across cellular compartments and physiological conditions. Previously, it was shown that serum starvation differentially affects the mRNA expression of individual isoforms, with DUT-N expression decreasing, DUT-M and DUT-3 increasing, while DUT-4 remaining unchanged in A549 and MDA-MB-231 cells. Moreover, the mRNA level of DUT-N is not altered upon serum starvation in HeLa cells, highlighting cell line-specific regulation of DUT-N mRNA expression. The presence of two nuclear isoforms may therefore provide additional flexibility in regulating nuclear dUTPase expression. The relatively stable expression of DUT-4 may potentially provide an alternative source of dUTPase activity when DUT-N expression is compromised. Thus, the presence of multiple dUTPase isoforms may reflect a combination of compartment-specific localization, differential regulation, and potential functional redundancy, although the specific contribution of each isoform remains to be investigated. Overall, this study provides a framework for understanding the spatial and temporal regulation of human dUTPase isoforms and facilitates future studies addressing their potential functions.

## Figures and Tables

**Figure 1:**
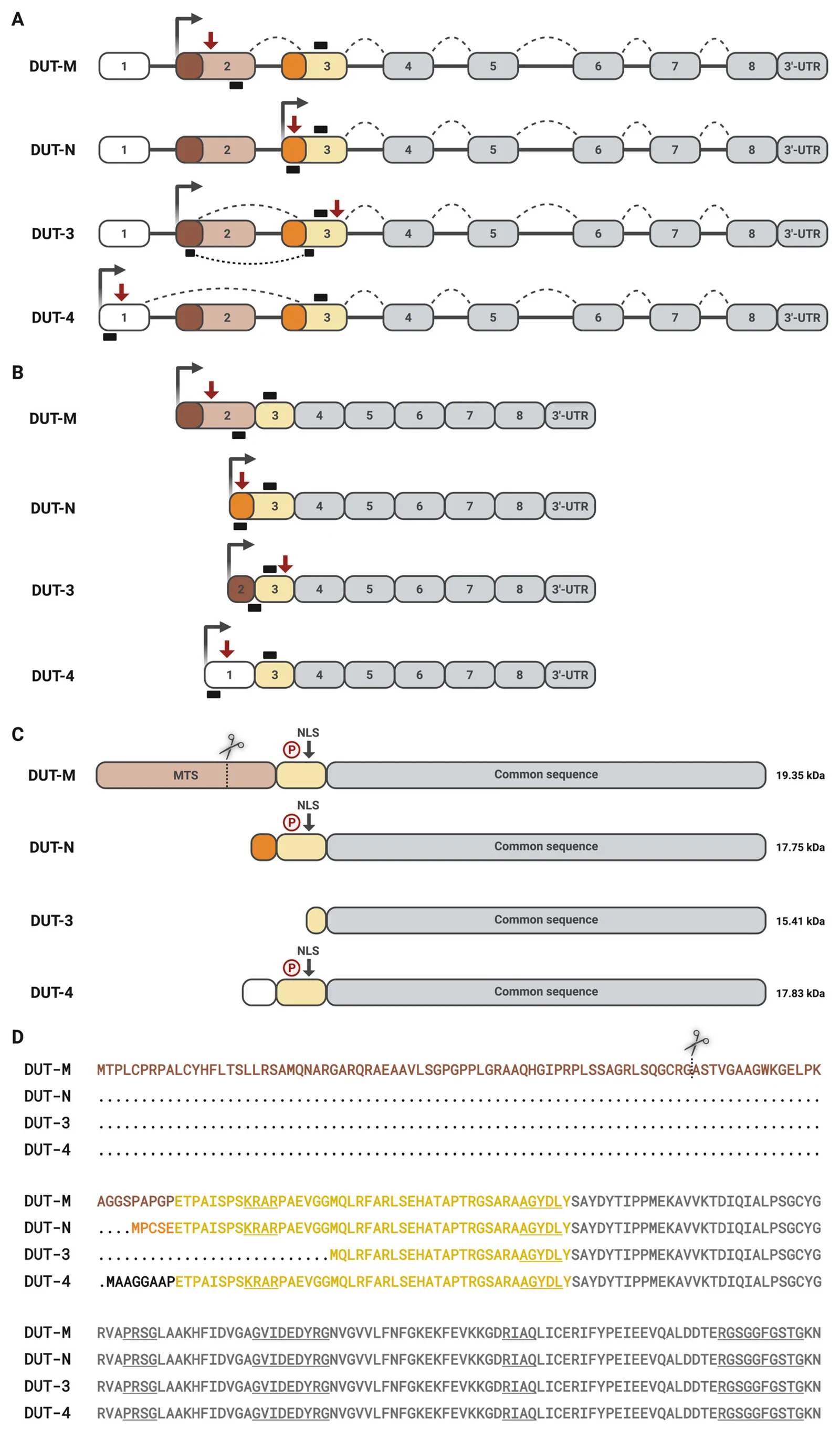
Schematic representation of alternative splicing in the *DUT* gene and the resulting transcripts and isoforms. (**A**) Schematic representation of the alternative splicing of *DUT* transcripts. Boxes indicate exons (1–8), while lines represent introns. Gray boxes indicate the common exons across all transcripts. Transcription start sites are marked by dark gray arrows, whereas translation initiation sites are indicated by red arrows. Dashed lines illustrate alternative splicing events. Small black boxes denote the positions of forward and reverse primers used for the analysis of isoform-specific mRNA expression of dUTPase. In the case of DUT-3, the target forward primer was designed to the exon-exon junction, indicated as a dotted line. Alternating shades of brown and orange indicate cryptic splice sites. (**B**) Schematic representation of mRNA transcripts generated by alternative splicing. Exons (1–8) are shown as boxes, with gray boxes indicating exons shared among all transcripts (4–8). Dark gray arrows indicate transcription start sites, while red arrows mark translation initiation sites. Small black boxes indicate the locations of the forward and reverse primers. (**C**) Schematic representation of dUTPase protein isoforms. The region encoded by exons common to all isoforms, referred to as the dUTPase core, is shown in light gray. DUT-M is the longest isoform and contains an N-terminal mitochondrial targeting signal (MTS, brown). Scissors represent the proteolytic cleavage site of DUT-M [6]. DUT-N, DUT-M, and DUT-4 isoforms contain a nuclear localization signal (NLS) [8] and a potential phosphorylation site indicated as a P (red) [9]. DUT-3 is the shortest isoform and lacks any localization signals. The theoretical molecular mass of each isoform is also shown. (**D**) Protein sequences of dUTPase isoforms. MTS of DUT-M is shown in brown; the N-terminal difference in DUT-N and DUT-4 is shown in orange and black, respectively. Scissors represent the proteolytic cleavage site of DUT-M [8]. The N-terminal part encoded by exon 3 is shared by DUT-M, DUT-N, and DUT-4, and is partially present in DUT-3, which is highlighted in yellow, while the common region is shown in gray. The NLS (KRAR) [8] and the five conserved motifs (gray) are underlined [1]. MTS, mitochondrial target sequence. NLS, nuclear localization signal. P, phosphorylation site. The figure was created in BioRender Vértessy, B. (2026) https://BioRender.com/l8ipno9.

**Figure 2:**
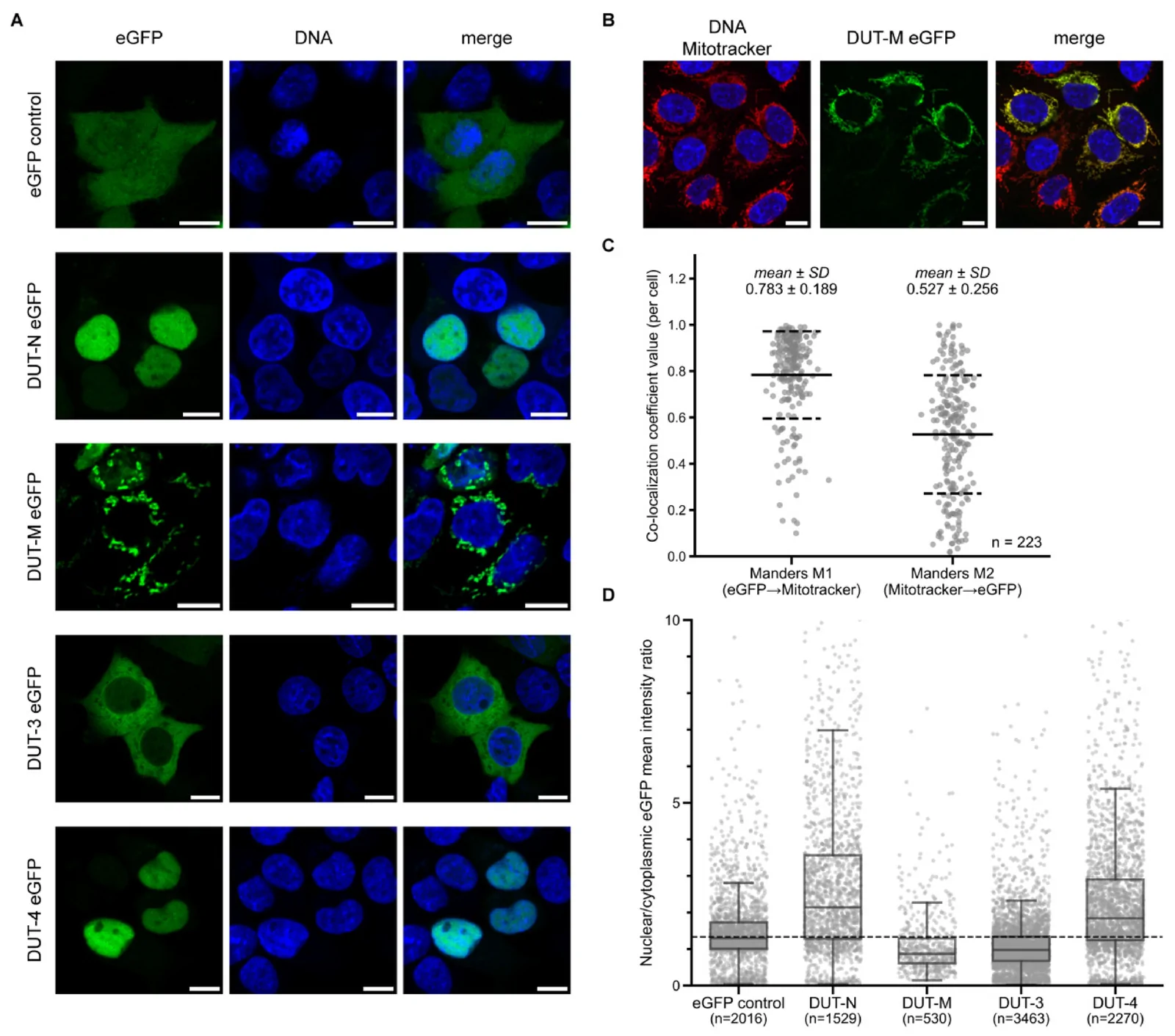
Subcellular localization of human dUTPase isoforms in HCT116 cells. (**A**) Confocal microscopy images show representative subcellular localization of eGFP-tagged constructs (control, DUT-N, DUT-M, DUT-3, and DUT-4) colored in green. DNA was stained with DAPI. Scale bar: 10 μm. (**B**) Mitochondrial localization of eGFP-tagged DUT-M isoform (green). Mitotracker displaying mitochondria, is shown in red. DNA was stained with DAPI. Scale bar: 10 μm. (**C**) Per-cell quantification of the co-localization between eGFP-tagged DUT-M and Mitotracker. The dot plot shows Manders’ M1 and M2 coefficients for each cell obtained from image analysis using CellProfiler. The mean and standard deviation for each co-localization value are shown at the top. *n* = 223 cells derived from two independent transfections. The M1 value reflects the fraction of the eGFP-labeled DUT-M signal overlapping with the Mitotracker signal, whereas M2 represents the fraction of the Mitotracker signal overlapping with the eGFP-tagged DUT-M signal. (**D**) Quantitative analysis of the subcellular localization of eGFP-tagged dUTPase isoforms and eGFP control. The box plot shows the distribution of nuclear/cytoplasmic eGFP mean intensity ratio values for each construct. Gray dots represent individual cells. n, number of analyzed cells for each construct derived from four independent transfections. Error bars indicate standard deviation. The dashed line shows the median nuclear/cytoplasmic eGFP mean intensity ratio of eGFP control cells (1.33), defined as the baseline. Whiskers show the most outlier data points within 1.5× the interquartile range of the first and third quartiles. Images were processed using Fiji, Cursor, and Adobe Illustrator.

**Figure 3:**
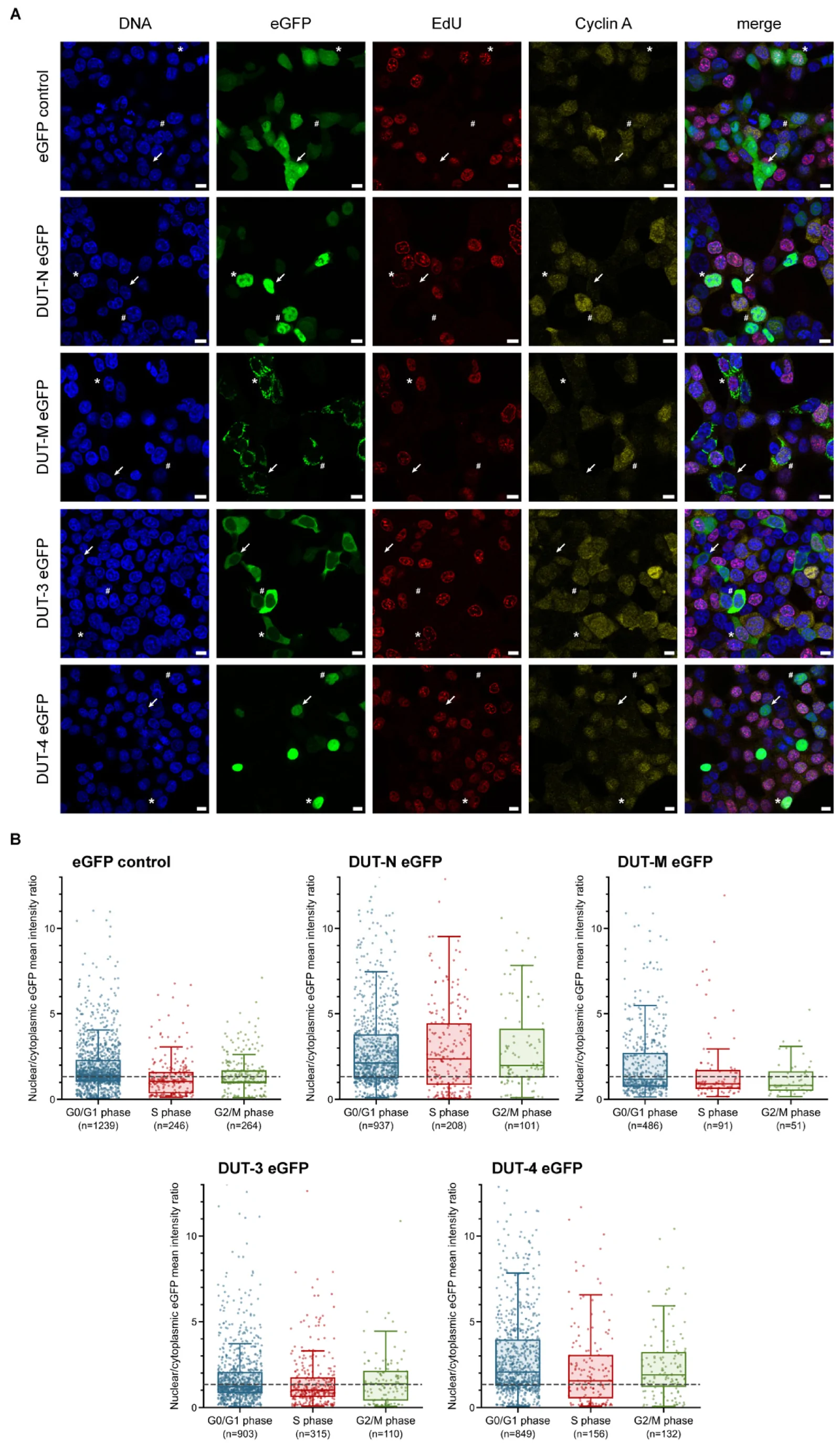
Cell cycle-associated localization of human dUTPase isoforms in HCT116 cells. (**A**) Confocal microscopy images show cell cycle-associated subcellular localization of eGFP-tagged constructs (control, DUT-N, DUT-M, DUT-3, and DUT-4) colored in green. Cell cycle phases, i.e., G0/G1, S, and G2/M, are distinguished by cyclin A (yellow) and EdU (red) staining. Arrows indicate cells in G0/G1 phase, asterisks show cells in S phase, while hash symbols indicate cells in G2/M phase according to our experimental setup. DNA was stained with DAPI. Scale bar: 10 μm. (**B**) Quantitative analysis of the subcellular localization of eGFP-tagged dUTPase isoforms and eGFP control. Box plots show the distribution of nuclear/cytoplasmic eGFP mean intensity ratio values for each construct and cell cycle phase (G0/G1 (blue), S (red), and G2/M (green)). Dots represent individual eGFP-positive cells included in the analysis. n, number of analyzed cells for each construct derived from three individual transfections. The dashed line shows the median nuclear/cytoplasmic eGFP mean intensity ratio of eGFP control cells (1.33), defined as the baseline. Boxes show the 25th–75th percentiles, and lines indicate the median values for each group. Whiskers show the most outlier data points within 1.5× the interquartile range of the first and third quartiles. Images were processed using Fiji, Cursor, and Adobe Illustrator.

**Figure 4:**
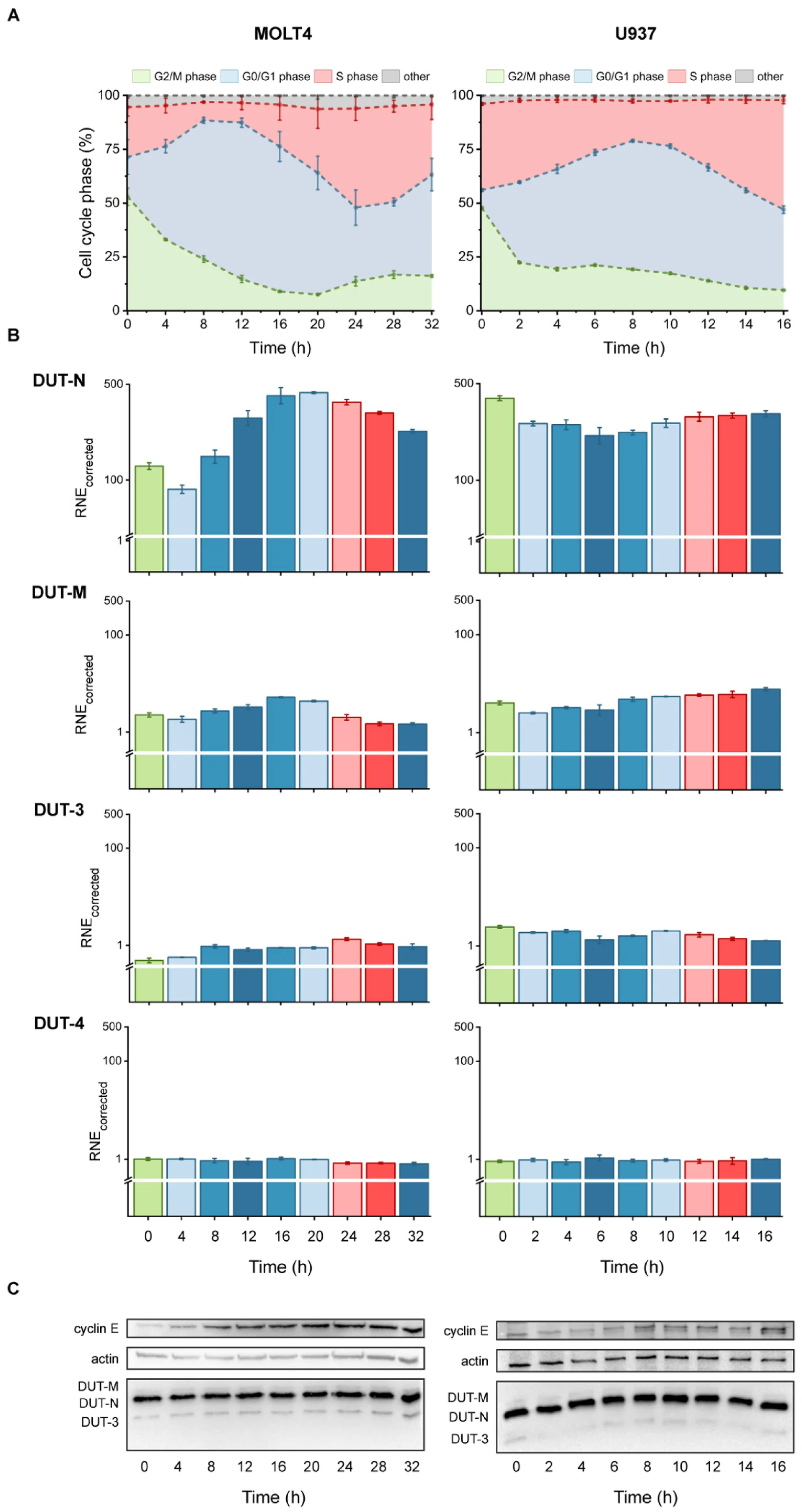
Cell cycle-associated expression of endogenous dUTPase isoforms in MOLT4 and U937 human leukemia cell lines. (**A**) Cells were collected every 4 h for 32 h in the case of MOLT4 cells and every 2 h for 16 h in the case of U937 cells. The distribution of cell cycle phases at each time point (G0/G1, blue; S, red; G2/M, green; and other, gray). (**B**) Relative, corrected, and normalized mRNA expression levels of dUTPase isoforms were determined across cell cycle phases in both cell lines (Supplementary Table S7). Columns are colored by cell cycle phase (G0/G1, blue; S, red; G2/M, green), with the corresponding population most enriched at each time point. Color intensity reflects the proportion of cells in each cell cycle phase. Expression levels are shown on a logarithmic scale. Error bars represent the standard error of the mean of mRNA expression across biological replicates (*n* = 3). Y-axis breaks are 10–40 for DUT-N, and 0.1–0.4 for DUT-M, DUT-3, and DUT-4. RNE-corrected, relative normalized corrected expression. (**C**) Western blot analysis to determine protein expression of human dUTPase isoforms in MOLT4 and U937 cell lines during the cell cycle. Original Western blot images are shown in Supplementary Figure S3. Figures were created using Origin 2018 and Adobe Illustrator.

## Data Availability

The original contributions presented in this study are included in the article/Supplementary Materials. Further inquiries can be directed to the corresponding authors.

## Supplementary Materials

The following supporting information can be downloaded at: https://www.mdpi.com/article/10.3390/biom16101385/s1. Supplementary information includes Tables S1–S7 and Supplementary Figures S1–S3. Table S1: Coding DNA sequences of DUT-N, DUT-M, DUT-3, and DUT-4 in the eGFP-N1 and -N3 vector. Table S2: Primer sequences generating the DUT-3 isoform by deleting the N-terminal region of DUT-N. Table S3: Primer sequences and their parameters used in this study for qPCR analysis. Bp, base pairs. Table S4: Correction factors used to adjust Cq values. Table S5: The average of Cq and corrected Cq values for each target and each biological replicate in the MOLT4 cell line. Table S6: The average of Cq and corrected Cq values for each target and each biological replicate in the U937 cell line. Table S7: The average of relative normalized corrected expression values across biological replicates, with lower and upper error bar values for each target and biological replicate. Figure S1: Cell cycle-associated relative normalized mRNA expression of endogenous dUTPase isoforms in U937 and MOLT4 cell lines was determined by RT-qPCR using CFX Maestro 2.0 software (Bio-Rad). Figure S2: Western blot analysis of eGFP-tagged dUTPase isoforms. HCT116 cells were transfected with C-terminal eGFP-tagged dUTPase constructs (DUT-N, DUT-M, DUT-3, DUT-4), eGFP alone, or non-transfected as a control. Figure S3: Uncropped Western blot images of human dUTPase isoforms during the cell cycle in MOLT4 and U937 cells.
