# Supplementary material for: Subcellular Localization and Cell Cycle-Associated Expression of Human dUTPase Isoforms
Source: Biomolecules. 2026 Sep 23;16(10):1385. doi: 10.3390/biom16101385 (PMC13702199; doi:10.3390/biom16101385)
Supplement: Supplementary file 1 [file biomolecules-16-01385-s001.zip › biomolecules-4494540-supplementary.pdf]

Supplementary Materials

Subcellular Localization and Cell Cycle-Associated Expression of Human dUTPase Isoforms

Otília Tóth [1,2], Gergely Attila Rácz [2], Eszter Oláh [2,3], Máté Tóth [1], Edit Szabó [2], György Várady [2], Gergely Róna [2,4], Beáta G. Vértessy [1,2,*] and Nikolett Nagy [2,*]

1 Department of Applied Biotechnology and Food Sciences, Faculty of Chemical Technology and Biotechnology, BME Budapest University of Technology and Economics, Műegyetem rkp. 3, H-1111 Budapest, Hungary; toth.otilia@ttk.hu (O.T.)
2 Institute of Molecular Life Sciences, HUN-REN Research Centre for Natural Sciences, Magyar tudósok körútja 2, H-1117 Budapest, Hungary
3 Doctoral School of Biology, Institute of Biology, ELTE Eötvös Loránd University, Pázmány Péter sétány 1/C, H-1117 Budapest, Hungary
4 Department of Biochemistry and Molecular Pharmacology, NYU Grossman School of Medicine, New York, NY 10016, USA
* Correspondence: vertessy.beata@ttk.hu (B.G.V.); nagy.nikolett@ttk.hu (N.N.)

Supplementary Table S1. Coding DNA sequences of DUT-N, DUT-M, DUT-3, and DUT-4 in the eGFP-N1 and -N3 vector.

Target	Coding DNA sequence (5′-3′)
**DUT-N**	ATGCCCTGCTCCGAGGAGACACCCGCCATCTCCCCTAGCAAGAGGGCCAGGCCTGCCGAGGTGGGCGGAATGCAGCTGA<br>GGTTTGCCAGACTGTCCGAGCACGCCACCGCCCCAACCAGAGGCAGCGCTAGGGCCGCTGGCTACGATCTGTACTCCGCC<br>TACGACTACACCATCCCTCCCATGGAGAAGGCCGTGGTGAAGACAGATATCCAGATCGCCCTGCCTTCCGGCTGCTACGG<br>CAGAGTGGCCCCCAGATCCGGCCTGGCTGCCAAGCACTTTATCGATGTGGGCGCCGGCGTGATCGACGAGGATTACAGA<br>GGCAATGTGGGCGTGGTGCTGTTCAACTTCGGCAAGGAGAAGTTTGAGGTGAAGAAGGGCGACAGAATCGCCCAGCTGA<br>TCTGCGAGAGGATCTTCTACCCCGAGATCGAGGAGGTGCAGGCCCTGGACGACACCGAGAGAGGCTCCGGCGGCTTCGG<br>CTCCACAGGCAAGAAT
**DUT-M**	ATGACTCCCCTCTGCCCTCGCCCCGCGCTCTGCTACCATTTCCTTACGTCTCTGCTTCGCTCAGCGATGCAAAACGCGCGA<br>GGCGCACGGCAGAGGGCCGAAGCCGCGGTACTCTCCGGGCCAGGCCCGCCCCTCGGCCGCGCCGCGCAGCACGGGATTC<br>CCCGGCCGCTGTCCAGCGCTGGCCGCCTGAGCCAAGGCTGCCGCGGAGCCAGTACAGTCGGGGCCGCTGGCTGGAAGGG<br>CGAGCTTCCTAAGGCGGGGGAAGCCCGGCGCCGGGGCCGGAGACACCCGCCATTTCACCCAGTAAGCGGGCCCGGCCT<br>GCGGAGGTGGGCGGCATGCAGCTCCGCTTTGCCCGGCTCTCCGAGCACGCCACGGCCCCCACCCGGGGCTCCGCGCGCG<br>CCGCGGGCTACGACCTGTACAGTGCCTATGATTACACAATACCACCTATGGAGAAAGCTGTTGTGAAAACGGACATTCAG<br>ATAGCGCTCCCTTCTGGGTGTTATGGAAGAGTGGCTCCACGGTCAGGCTTGGCTGCAAAACACTTTATTGATGTAGGAGCT<br>GGTGTCATAGATGAAGATTATAGAGGAAATGTTGGTGTTGTACTGTTTAATTTTGGCAAAGAAAAGTTTGAAGTCAAAAA<br>AGGTGATCGAATTGCACAGCTCATTTGCGAACGGATTTTTTATCCAGAAATAGAAGAAGTTCAAGCCTTGGATGACACCG<br>AAAGGGGTTCAGGAGGTTTTGGTTCCACTGGAAAGAATTAAGGTACC
**DUT-3**	ATGCAGCTGAGGTTTGCCAGACTGTCCGAGCACGCCACCGCCCCAACCAGAGGCAGCGCTAGGGCCGCTGGCTACGATC<br>TGTACTCCGCCTACGACTACACCATCCCTCCCATGGAGAAGGCCGTGGTGAAGACAGATATCCAGATCGCCCTGCCTTCC<br>GGCTGCTACGGCAGAGTGGCCCCCAGATCCGGCCTGGCTGCCAAGCACTTTATCGATGTGGGCGCCGGCGTGATCGACGA<br>GGATTACAGAGGCAATGTGGGCGTGGTGCTGTTCAACTTCGGCAAGGAGAAGTTTGAGGTGAAGAAGGGCGACAGAATC<br>GCCCAGCTGATCTGCGAGAGGATCTTCTACCCCGAGATCGAGGAGGTGCAGGCCCTGGACGACACCGAGAGAGGCTCCG<br>GCGGCTTCGGCTCCACAGGCAAGAAT
**DUT-4**	ATGGCTGCTGGAGGAGCTGCTCCTGAAACACCAGCTATCTCCCCCAGCAAGAGGGCTAGACCAGCTGAAGTGGGCGGAA<br>TGCAGCTGAGATTTGCCAGGCTGTCCGAGCACGCTACAGCTCCTACAAGAGGCAGCGCTAGAGCCGCTGGATACGATCTG<br>TACTCCGCCTACGATTACACAATCCCCCCCATGGAGAAGGCCGTGGTTAAGACAGATATCCAGATCGCCCTGCCCAGCGG<br>ATGTTACGGAAGAGTTGCCCCAAGGAGCGGCCTGGCTGCTAAGCATTTTATCGATGTGGGCGCCGGCGTGATCGATGAAG<br>ATTACAGAGGCAACGTGGGCGTGGTGCTGTTTAATTTTGGCAAGGAGAAGTTTGAGGTGAAGAAGGGCGACAGGATCGC<br>CCAACTGATCTGCGAAAGAATCTTCTACCCCGAGATCGAGGAGGTGCAGGCTCTGGATGACACCGAAAGAGGCAGCGGA<br>GGATTTGGCAGCACCGGAAAAAACGCGGTACC

Supplementary Table S2. Primer sequences generating the DUT-3 isoform by deleting the N-terminal region of DUT-N.

	Primer sequence (5′-3′)
**Fw**	ATGCAGCTGAGGTTTGCCAGACTG
**Rev**	CTCGAGATCTGAGTCCGGTAGCGCTAG

Supplementary Table S3. Primer sequences and their parameters used in this study for qPCR analysis. Bp, base pairs.

Target symbol	Primer sequences (5′-3′)	Length of PCR product (bp)	T_m of PCR product (°C)	Primer design	PCR efficiency (%)
DUT-N	Fw: TCTCCTCGCTCGCCTTCTG<br>Rev: CCCGCTTACTGGGTGAAATGG	73	83.5	Exonic	97.0
DUT-M	Fw: GCTGGCTGGAAGGGCGAG<br>Rev: CCCGCTTACTGGGTGAAATGG	85	88.0	Intron-flanking	96.6
DUT-3	Fw: GCACGTCCGGAGAGACACC<br>Rev: CCCGCTTACTGGGTGAAATGG	42	79.4	Intron-flanking	99.3
DUT-4	Fw: ACTCAGAGCCCGGGAGTC<br>Rev: CCCGCTTACTGGGTGAAATGG	73	86.3	Intron-flanking	96.4
CNOT4	Fw: GTCCAAAACCTGACTGCATGTATC<br>Rev: GGTGTTTACCCGCCTGCAT	87	80.8	Intron-spanning	96.3
TBP	Fw: ATATAATCCCAAGCGGTTTGCTG<br>Rev: AAAATCAGTGCCGTGGTTCG	66	79.8	Intron-spanning	97.4
PUM1	Fw: TGCGGGAGATTGCTGGACAT<br>Rev: GTGTGGCACGCTCCAGTTTC	87	80.4	Intron-flanking	98.4
SNW1	Fw: GCAGCTCCTGATAAGAGGTCG<br>Rev: CCGAGGATTAGGAACACCGAG	87	78	Intron-spanning	95.8
UBC	Fw: GGTCGCAGTTCTTGTTTGTGG<br>Rev: TTCACGAAGATCTGCATTGTCAAG	60	78.4	Exonic	100.7

Supplementary Table S4. Correction factors used to adjust Cq values.

Target symbol	Correction factor
DUT-N	0.9761
DUT-M	0.9668
DUT-3	1.0104
DUT-4	1.0000

Supplementary Table S5. The average of Cq and corrected Cq values for each target and each biological replicate in the MOLT4 cell line.

		DUT-N		*DUT-M*		*DUT-3*		*DUT-4*	
**Time point (hour)**	**Biological replicate**	**Average of Cq values**	**Average of corrected Cq values**	**Average of Cq values**	**Average of corrected Cq values**	**Average of Cq values**	**Average of corrected Cq values**	**Average of Cq values**	**Average of corrected Cq values**
**0**	1	25.59	24.98	32.36	31.29	33.17	33.51	33.16	33.16
**0**	2	26.09	25.47	33.18	32.07	33.85	34.20	33.15	33.15
**0**	3	26.11	25.49	32.80	31.71	33.93	34.28	33.39	33.39
**4**	1	25.82	25.20	32.84	31.75	33.32	33.67	33.12	33.12
**4**	2	26.20	25.58	32.44	31.36	33.43	33.77	33.08	33.08
**4**	3	26.14	25.51	33.00	31.90	33.37	33.71	32.98	32.98
**8**	1	25.95	25.34	33.27	32.16	33.67	34.02	33.86	33.86
**8**	2	26.43	25.80	32.80	31.71	33.37	33.72	33.40	33.40
**8**	3	25.99	25.37	32.79	31.70	33.35	33.70	33.53	33.53
**12**	1	25.43	24.82	32.78	31.69	33.57	33.91	33.83	33.83
**12**	2	25.82	25.21	32.89	31.80	33.52	33.87	33.57	33.57
**12**	3	25.19	24.58	32.27	31.20	33.17	33.51	33.00	33.00
**16**	1	25.12	24.52	32.33	31.26	33.41	33.75	33.47	33.47
**16**	2	25.20	24.60	31.96	30.90	33.01	33.36	33.12	33.12
**16**	3	25.19	24.59	32.53	31.45	33.56	33.91	33.41	33.41
**20**	1	25.08	24.48	32.24	31.17	33.35	33.70	33.27	33.27
**20**	2	24.83	24.24	32.17	31.10	32.94	33.28	33.14	33.14
**20**	3	25.05	24.45	32.36	31.29	33.27	33.62	33.25	33.25
**24**	1	25.80	25.18	33.14	32.04	33.26	33.60	34.00	34.00
**24**	2	25.60	24.99	33.30	32.19	33.50	33.85	33.75	33.75
**24**	3	25.54	24.93	33.72	32.60	33.51	33.86	33.91	33.91
**28**	1	25.76	25.14	33.66	32.54	33.63	33.98	33.82	33.82
**28**	2	26.03	25.40	34.08	32.95	33.71	34.06	34.27	34.27
**28**	3	25.74	25.12	33.26	32.15	33.59	33.93	33.74	33.74
**32**	1	26.10	25.48	33.80	32.68	34.06	34.42	34.15	34.15
**32**	2	26.14	25.52	33.85	32.73	33.82	34.17	34.02	34.02
**32**	3	26.21	25.59	33.62	32.50	33.55	33.90	33.97	33.97

Supplementary Table S6. The average of Cq and corrected Cq values for each target and each biological replicate in the U937 cell line.

		DUT-N		*DUT-M*		*DUT-3*		*DUT-4*	
**Time point (hour)**	**Biological replicate**	**Average of Cq values**	**Average of corrected Cq values**	**Average of Cq values**	**Time point (hour)**	**Biological replicate**	**Average of Cq values**	**Average of corrected Cq values**	**Average of Cq values**
**0**	1	24.47	23.89	30.84	0	1	24.47	23.89	30.84
**0**	2	24.51	23.92	31.37	0	2	24.51	23.92	31.37
**0**	3	24.33	23.75	30.66	0	3	24.33	23.75	30.66
**2**	1	24.51	23.92	30.86	2	1	24.51	23.92	30.86
**2**	2	24.46	23.88	31.06	2	2	24.46	23.88	31.06
**2**	3	24.39	23.81	31.12	2	3	24.39	23.81	31.12
**4**	1	24.63	24.04	30.87	4	1	24.63	24.04	30.87
**4**	2	24.60	24.01	31.04	4	2	24.60	24.01	31.04
**4**	3	24.50	23.91	30.90	4	3	24.50	23.91	30.90
**6**	1	24.53	23.94	30.87	6	1	24.53	23.94	30.87
**6**	2	24.73	24.14	31.32	6	2	24.73	24.14	31.32
**6**	3	24.37	23.78	30.38	6	3	24.37	23.78	30.38
**8**	1	24.62	24.03	30.51	8	1	24.62	24.03	30.51
**8**	2	24.91	24.32	31.25	8	2	24.91	24.32	31.25
**8**	3	24.87	24.28	30.55	8	3	24.87	24.28	30.55
**10**	1	24.83	24.24	31.02	10	1	24.83	24.24	31.02
**10**	2	24.80	24.21	30.71	10	2	24.80	24.21	30.71
**10**	3	24.90	24.31	30.77	10	3	24.90	24.31	30.77
**12**	1	24.65	24.06	30.77	12	1	24.65	24.06	30.77
**12**	2	24.82	24.23	30.84	12	2	24.82	24.23	30.84
**12**	3	24.46	23.87	30.37	12	3	24.46	23.87	30.37
**14**	1	24.34	23.76	30.39	14	1	24.34	23.76	30.39
**14**	2	24.31	23.73	30.01	14	2	24.31	23.73	30.01
**14**	3	24.60	24.01	30.87	14	3	24.60	24.01	30.87
**16**	1	24.40	23.82	30.14	16	1	24.40	23.82	30.14
**16**	2	24.28	23.70	30.16	16	2	24.28	23.70	30.16
**16**	3	24.42	23.84	30.36	16	3	24.42	23.84	30.36

Supplementary Table S7. The average of relative normalized corrected expression values across biological replicates, with lower and upper error bar values for each target and biological replicate.

		MOLT4 cell line			U937 cell line		
**Time point (hour)**	**Target**	**Average of relative normalized corrected expression**	**Lower error**	**Upper error**	**Average of relative normalized corrected expression**	**Lower error**	**Upper error**
**0**	***DUT-N***	126.60	119.51	134.11	391.42	375.17	408.37
**4**	***DUT-N***	85.88	80.09	92.10	256.93	247.55	266.67
**8**	***DUT-N***	148.61	133.22	165.77	252.06	232.83	272.89
**12**	***DUT-N***	283.85	250.95	321.05	210.31	183.72	240.76
**16**	***DUT-N***	412.96	360.51	473.03	221.84	212.91	231.15
**20**	***DUT-N***	434.32	428.05	440.68	258.81	241.53	277.33
**24**	***DUT-N***	370.08	354.42	386.42	287.83	266.85	310.46
**28**	***DUT-N***	308.66	301.64	315.85	293.30	281.36	305.76
**32**	***DUT-N***	227.22	220.03	234.66	302.56	287.74	318.14
**0**	***DUT-M***	2.26	2.05	2.50	4.06	3.71	4.45
**4**	***DUT-M***	1.84	1.61	2.11	2.53	2.44	2.63
**8**	***DUT-M***	2.73	2.49	2.99	3.24	3.06	3.42
**12**	***DUT-M***	3.27	2.90	3.69	2.89	2.26	3.70
**16**	***DUT-M***	5.20	5.08	5.33	4.81	4.39	5.28
**20**	***DUT-M***	4.37	4.21	4.54	5.49	5.42	5.57
**24**	***DUT-M***	2.01	1.76	2.30	5.85	5.50	6.21
**28**	***DUT-M***	1.49	1.37	1.62	6.03	5.18	7.01
**32**	***DUT-M***	1.47	1.39	1.57	7.66	7.09	8.28
**0**	***DUT-3***	0.49	0.44	0.55	2.45	2.28	2.62
**4**	***DUT-3***	0.57	0.56	0.57	1.88	1.81	1.96
**8**	***DUT-3***	0.96	0.89	1.03	2.02	1.88	2.17
**12**	***DUT-3***	0.82	0.76	0.89	1.33	1.11	1.59
**16**	***DUT-3***	0.90	0.88	0.91	1.60	1.54	1.66
**20**	***DUT-3***	0.89	0.85	0.94	2.03	2.00	2.07
**24**	***DUT-3***	1.34	1.25	1.43	1.69	1.52	1.87
**28**	***DUT-3***	1.07	1.01	1.13	1.40	1.30	1.51
**32**	***DUT-3***	0.94	0.83	1.07	1.27	1.25	1.29
**0**	***DUT-4***	1.01	0.94	1.09	0.92	0.86	0.97
**4**	***DUT-4***	1.01	0.98	1.04	0.98	0.91	1.05
**8**	***DUT-4***	0.93	0.84	1.04	0.88	0.78	0.99
**12**	***DUT-4***	0.90	0.77	1.05	1.07	0.93	1.24
**16**	***DUT-4***	1.04	0.97	1.11	0.95	0.88	1.02
**20**	***DUT-4***	0.99	0.97	1.01	0.97	0.91	1.04
**24**	***DUT-4***	0.83	0.79	0.88	0.91	0.83	1.00
**28**	***DUT-4***	0.83	0.80	0.86	0.93	0.80	1.09
**32**	***DUT-4***	0.80	0.75	0.86	1.01	0.98	1.04

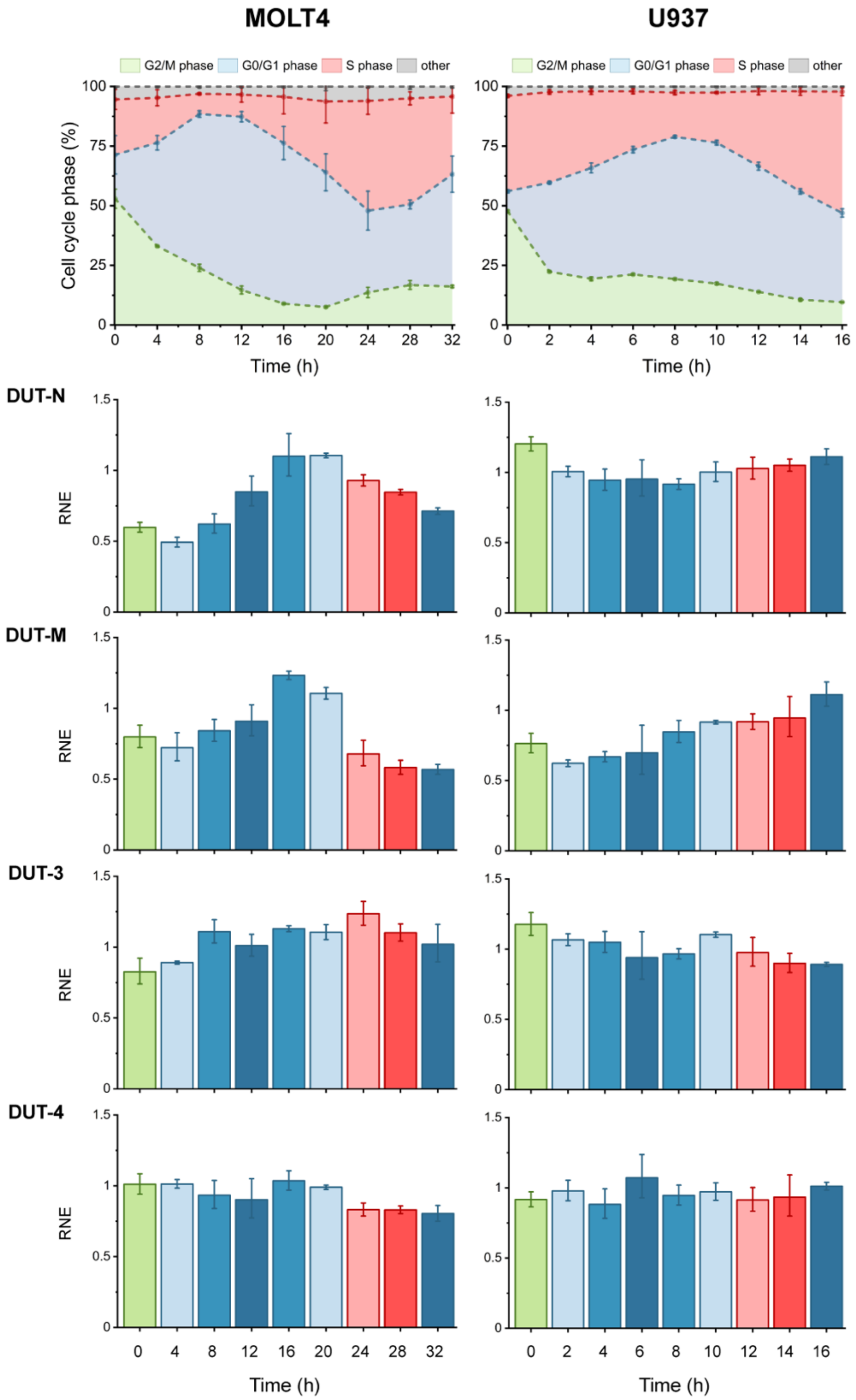

Supplementary Figure S1. Cell cycle-associated relative normalized mRNA expression of endogenous dUTPase isoforms in U937 and MOLT4 cell lines was determined by RT-qPCR using CFX Maestro 2.0 software (Bio-Rad). Graphs display relative normalized expression (RNE) values relative to zero for each target. Cells were collected every 4 hours for 32 hours in the case of MOLT4 cells and every 2 hours for 16 hours in the case of U937. The distribution of cell cycle phases at each time point (G0/G1, blue; S, red; G2/M, green; and other, gray) is shown at the top. Error bars represent the standard error of the mean of mRNA expression across biological replicates (n=3) and technical replicates (n=3). The image was created with Origin 2018 and Adobe Illustrator.

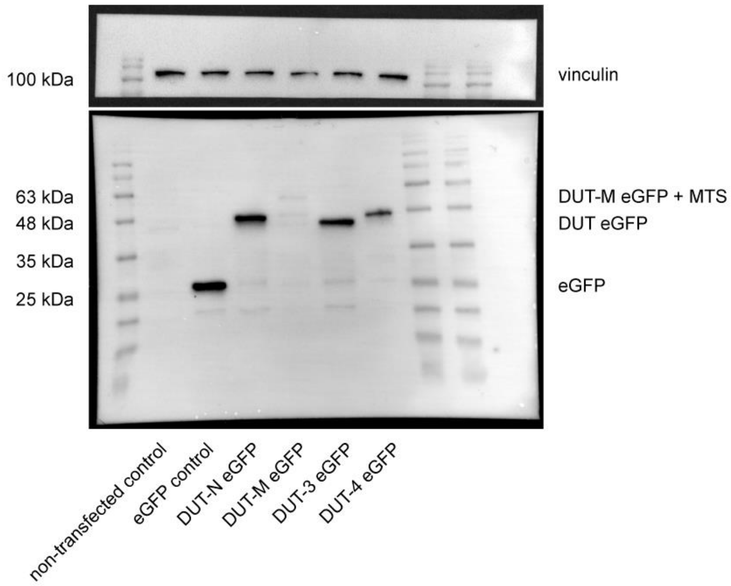

Supplementary Figure S2. Western blot analysis of eGFP-tagged dUTPase isoforms. HCT116 cells were transfected with C-terminal eGFP-tagged dUTPase constructs (DUT-N, DUT-M, DUT-3, DUT-4), eGFP alone, or non-transfected as a control. Transfected cells were lysed in RIPA buffer, and protein expression was analyzed by Western blotting, immunostained with antibodies against eGFP to mark controls and dUTPase constructs, and vinculin as a loading control. The expected fusion proteins, together with a small amount of free eGFP, were detected in all samples transfected with DUT eGFP constructs. The image was created with ImageLab 4.1 (Bio-Rad) and Adobe Illustrator.

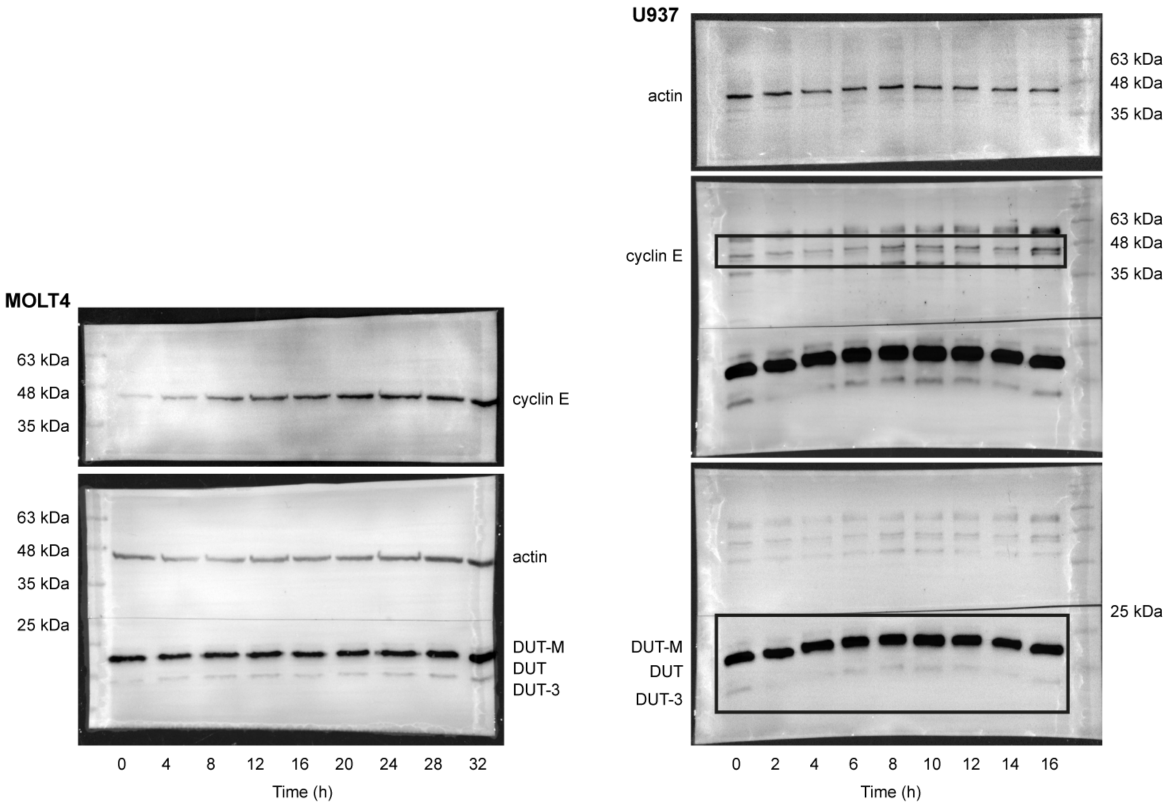

Supplementary Figure S3. Uncropped Western blot images of human dUTPase isoforms during the cell cycle in MOLT4 and U937 cells. For U937 cells, the boxed bands of dUTPase and cyclin E indicate the bands shown in Figure 4. The sizes of the protein ladder bands are indicated. The image was created with ImageLab 4.1 (Bio-Rad) and Adobe Illustrator.
